# Supplementary material for: Differential Effects of Platelet Factor 4 (CXCL4) and Its Non-Allelic Variant (CXCL4L1) on Cultured Human Vascular Smooth Muscle Cells
Source: Int J Mol Sci. 2022 Jan 6;23(2):580. doi: 10.3390/ijms23020580 (PMC8775478; doi:10.3390/ijms23020580)
Supplement: Supplementary file 1 [file ijms-23-00580-s001.zip › ijms-1503888-supplementary.pdf]

Differential effects of platelet factor 4 (CXCL4) and its non-allelic variant (CXCL4L1) on cultured human vascular smooth muscle cells

Dawid M. Kaczor, Rafael Kramann, Tilman M. Hackeng, Leon J. Schurgers, Rory R. Koenen

Supplementary material

Detailed methods

Reagents

CXCL4 was isolated from expired platelet packs using a method described elsewhere [1]. Recombinant CXCL4L1 was expressed in E. coli and purified in our laboratory similar as described previously [2,3] and analyzed by Xevo® UPLC-MS (Waters Corporation, Milford, MA) as described [4]. Receptor-associated protein (RAP) was purchased from Enzo Life Sciences (Lörrach, Germany). Native human LDL (SAE0053), Dynasore (CAS: 304448-55-3) and PitStop2 (CAS: 1419093-54-1) and TRI reagent were purchased from Merck Millipore (Darmstadt, Germany). Interleukin 6 (IL) ELISA kits, 1,1'-dioctadecyl-3,3,3',3'-tetramethylindocarbocyanine perchlorate (DiI)-labeled oxLDL, 2',7'-dichlorofluorescin diacetate (DCFDA), and attachment factor (AF), anti-DARC clone 2C3, polyclonal anti-CXCL4L1 antibody, and goat anti-rabbit antibodies conjugated with AF532 were purchased from ThermoFisher Scientific (Waltham, MA). Native human LDL was labeled with DiI as described [5]. Cytokines and rabbit anti-human CXCL4 were from Peprotech (Rocky Hill, NJ). Monoclonal mouse anti-CXCR3 clone # 49801 and monoclonal mouse anti-DARC, clone #358307 were from R&D systems (Minneapolis, MN). FITC-conjugated goat anti-mouse from Jackson ImmunoResearch (Ely, UK). MicroBCA kit and iScript™ Reverse Transcription Supermix were from Bio-Rad (Hercules, CA). Takyon™ No Rox SYBR® MasterMix dTTP blue was from Eurogentec (Seraing, Belgium). O-cresolphthalein assay was purchased from Randox (Crumlin, UK).

Cell culture

Human vascular smooth muscle cells (VSMCs) were isolated in our laboratory from tissue explants (human thoracic aorta) as described previously [6,7]. Collection, storage, and use of tissue and human aortic samples were performed in agreement with the Dutch Code for Proper Secondary Use of Human Tissue. Human VSMCs were cultured in DMEM medium supplemented with 20% FCS, 2 mM L-glutamine, 1% penicillin/streptomycin and incubated in a humidified atmosphere with 5% CO_2 at 37°C. Importantly, in all experiments VSMCs (between passage 4 and 10) isolated from a single patient were used. Before the start of experiments cells were cultured to at least 80% confluence.

CXCL4/CXCL4L1 internalization

Before the experiment, all culture plates were coated with AF. Human VSMC were seeded in a black 96-well plate at a density of $3\text{-}5\times10^3$ cells/well and incubated in DMEM, supplemented with 20% FCS overnight. The next day, cells were starved in DMEM with 0.5% FCS overnight. After that, cells were incubated with vehicle (50 mM Na-Acetate pH 5.5, 600 mM NaCl and 0.5 mM EDTA), 0.5 μg/ml CXCL4 or CXCL4L1 in DMEM containing 0.5% FCS for 1 hour, either at 37°C or 4°C.

In chemokine receptor blocking experiments, 10 μg/ml of blocking antibodies (anti-CXCR3 or anti-DARC clones #358307 and 2C3) for up to 30 minutes, and then 0.5 μg/ml of the chemokines (CXCL4 or CXCL4L1) were added to the cells and the culture plates were incubated for additional 1 hour at 37°C.

In LDL receptor family blocking experiments, the cells were starved in DMEM supplemented with 0.3% BSA overnight (to prevent the interference of lipids from FCS). The following day, the cells were pre-treated with vehicle or RAP (200 nM) for 30 minutes. Subsequently, the cells were incubated with vehicle, DiI-nLDL (5 μg/ml) alone or RAP and DiI-nLDL together for additional 1 hour at 37°C.

In endocytosis blocking experiments, the cells were pre-treated with Dynasore (80 μM) or PitStop2 (15 μM) for 15-30 minutes. Then, the fresh medium (DMEM + 0.3% BSA) containing

Dynasore (80 µM) or PitStop2 (15 µM) together with CXCL4 or CXCL4L1 (0.5 µg/ml) was added to the cells and the culture plates were incubated for additional 1 hour at 37°C.

For oxidized LDL coincubation experiments, the cells were simultaneously treated with (DiI)-labeled oxLDL (5 µg/ml) and vehicle, CXCL4 (0.5 µg/ml) or CXCL4L1 (0.5 µg/ml) in DMEM containing 0.3% BSA for 1 hour at 37°C.

In experiments exploring the influence of VSMCs phenotype, cells were pre-incubated with nothing (control), PDGF (20 ng/ml, 3 days) or heparin (200 U/ml, 5 days) in DMEM with 20% FCS or cultured in low serum DMEM (0.5% FCS). After up to 5 days, cells were starved in DMEM with 0.5% FCS overnight before addition of the chemokines (0.5 µg/ml).

Then, the cells were washed with 200 U/ml heparin to remove the remaining chemokines from the cell surface. After that, cells were fixed with 4% paraformaldehyde and blocked with blocking buffer (PBS containing 2% BSA) with or without 0.1% Triton X-100 for 1 hour at room temperature. The cells were stained as described below.

LDL receptor family blockade by RAP or endocytosis inhibitors

Before the experiment, all culture plates were coated with the AF. Human VSMCs were seeded in a black 96-well plate at a density of 5.000 cells/well and incubated in DMEM, supplemented with 20% FCS for 24 hours. The next day, the cells were starved in DMEM supplemented with 0.3% BSA overnight (to prevent the interference of lipids from FCS). The following day, the cells were pre-treated with vehicle or RAP (200 nM) for 30 minutes. Subsequently, the cells were incubated with vehicle, DiI-nLDL (5 µg/ml) alone or RAP and DiI-nLDL together for additional 1 hour at 37°C.

In endocytosis blocking experiments, the cells were pre-treated with Dynasore (CAS: 304448-55-3) (80 µM) or PitStop2 (CAS: 1419093-54-1) (15 µM) for 15-30 minutes. Then, the fresh medium (DMEM + 0.3% BSA) containing Dynasore (80 µM) or PitStop2 (15 µM) together with DiI-nLDL (5 µg/ml) was added to the cells and the culture plates were incubated for additional 1 hour at 37°C.

Afterwards, the cells were incubated with 200 U/ml heparin for 5 minutes. Subsequently, cells were fixed with 4% paraformaldehyde for 10 minutes at room temperature. Next, cells were washed with PBS and nuclei were stained with Hoechst solution. Then, the cell count and the fluorescence were analysed with Cytation™ (BioTek, Agilent, Santa Clara, Ca)

Immunocytochemistry

The primary antibody (rabbit anti-human CXCL4 from Peprotech or anti-CXCL4L1 from ThermoFischer) was added to VSMCs at a final concentration of 2 µg/ml and incubated overnight at 4°C. After washing, the secondary antibody was added (goat anti-rabbit, conjugated with AF532 from Life Technologies) at a final concentration of 5 µg/ml for 1 hour. Next, cells were washed and nuclei were stained with Hoechst solution. Then, the cell count and the fluorescence was analysed with Cytation™. In negative control wells, the addition of primary antibody was omitted. The microscopic pictures were taken with Cytation™. Cell counting and fluorescence analysis were performed with ImageJ software.

Chemokine receptor staining - Then, the primary antibody (anti-CXCR3 or anti-DARC clone #358307) was added at a final concentration of 10 µg/ml and incubated overnight at 4°C. After washing, the secondary antibody was added (FITC-conjugated goat anti-mouse from Jackson) at a final concentration of 6 µg/ml for 1 hour. Next, cells were washed, and nuclei were stained with Hoechst solution. Then, the cell count and the fluorescence were analyzed with Cytation™. In negative control wells, the addition of primary antibody was omitted.

Quantitative real-time PCR (qPCR)

Primary VSMCs were seeded in 6-well plates at a density of 120.000 cells/well in DMEM with 20% FBS. The next day cells were treated with vehicle, CXCL4 (1 µg/ml) or CXCL4L1 (1 µg/ml) in DMEM, supplemented with 2.5% FBS and kept in the incubator for 24 or 72 hours. After that, VSMCs were directly lysed in TRI reagent (1 ml/well) and total RNA was extracted using a manufacturer's protocol. RNA concentration was quantified spectrophotometrically at 260 nm using NanoDrop™. Reverse Transcription was performed using iScript™ Reverse Transcription Supermix for RT-qPCR, following manufacturer's recommendations. Gene expression levels were quantified by real-time quantitative PCR (qPCR) on a LightCycler 480 Real-Time PCR instrument (Roche Applied Science, Basel, Switzerland). Amplification reactions were carried out in a volume of 10 µl including 45 ng of total cDNA, 5.5 µl of Takyon™ No Rox SYBR® MasterMix dTTP blue and 62.5 nM of each primer. Relative quantification was calculated by plotting Cq ratios. Experiments were performed in triplicate, with internal triplicate determinations.

Table S1. Primer sequences

Target	Sequence
**GAPDH**	Forward: AAC-GGA-TTT-GGT-CGT-ATT-GGG-C
	Reverse: CTT-GAC-GGT-GCC-ATG-GAA-TTT-G
**CNN1**	Forward: GCT-GGA-GAA-CAT-CGG-CAA-CTT-CAT-CAA-G
	Reverse: GCT-CCT-GCT-TCT-CTG-CGT-ACT-TCA-CTC
**α-SMA**	Forward: CCT-GAC-TGA-GCG-TGG-CTA-TT
	Reverse: GCC-CAT-CAG-GCA-ACT-CGT-AA
**IL-1β**	Forward: AAA-CCT-CTT-CGA-GGC-ACA-AG
	Reverse: GTT-TAG-GGC-CAT-CAG-CTT-CA
**IL-6**	Forward: ACA-TCC-TCG-ACG-GCA-TCT-CA
	Reverse: TCA-CCA-GGC-AAG-TCT-CCT-CAT
**KLF4**	Forward: AGA-GGA-GCC-CAA-GCC-AAA
	Reverse: AGC-CGT-CCC-AGT-CAC-AGT
**NLRP3**	Forward: GGG-ACT-GAA-GCA-CCT-GTT-GT
	Reverse: GAG-TCT-GGT-CAG-GGA-ATG-GC

Proliferation assay using the xCELLigence system

To measure the proliferation rate of VSMCs, an in vitro xCELLigence RTCA (real-time cell analysis, Agilent) platform was used. Cells were seeded into 96-well electronic microtiter plates (E-plate®) at 3000 cells/well in DMEM and observed for 9 days. Adhesion of cells to the gold microelectrodes in the wells impedes the flow of electric current between electrodes and impedance value is plotted as a unitless parameter (Cell Index) over course of time. The rate of cell growth was determined by calculating the slope of the curve line between the end of the lag phase (0-50 hours) and the start of the stationary confluent phase (after 100 hours).

Calcification assay

Primary VSMCs were seeded in the 48-well plates (10.000 cells/well). After overnight incubation in DMEM (supplemented with 20% FCS) cells were treated with control medium (DMEM), increased calcium (2.7 mM) and phosphate (2.5 mM) or calcium/phosphate in combination with CXCL4 or CXCL4L1 (1 µg/ml or 10 µg/ml). To accelerate calcification, serum was reduced to 0.5% FCS in all conditions. Purified, plasma CXCL4 or recombinant CXCL4L1 was added to calcium/phosphate enriched cell culture media and incubated for 24 hours. After the experiment, calcification was quantified. Briefly, calcified material from each well was extracted from non-fixed cell layers with 0.1 mol/l HCl, incubated for 30 minutes at room temperature and analysed using o-cresolphthalein assay according to manufacturer's recommendations. Hereafter, the remaining solution was neutralized with 0.1 M NaOH and 0.2% SDS. Subsequently, protein determination was performed by MicroBCA kit. At the end the level of calcification was normalized to protein content.

Reactive oxygen species (ROS) assay

Human VSMCs were seeded in a black 96-well plate at a density of 8.000 cells/well and incubated in DMEM, supplemented with 2.5% FCS overnight. The next day, cells were pre-incubated with the dye for 30 min at 37°C and stimulated with vehicle and various test media at times of measurement. Hydrogen peroxide (H_2O_2) was used as a positive control. The combination of calcium (2.7 mM) and phosphate (2.5 mM) alone or combined with CXCL4 or CXCL4L1 [10 µg/ml] was used. All mastermixes were prepared in the KRPG medium, supplemented with Hoechst solution to visualize nuclei. Intracellular reactive oxygen species (ROS) were measured using cell permeable reagent DCFDA, a fluorogenic dye that measures hydroxyl, peroxyl and other ROS activity in the cell. The fluorescence intensity was detected with Cytation™ for 6 hours in controlled conditions (5% CO_2, 37°C). The data was analyzed by calculating area under the curve (AUC) and normalized to the cell count.

Statistical analysis

Statistical analysis was performed using Graphpad Prism 9.0.0. Data are presented as means ± SD and were compared by (non-)parametric 1-way or 2-way ANOVA. Appropriate correction for multiple comparisons was achieved depending on the statistical test. Differences with $P<0.05$ were considered as statistically significant. Each experiment was independently repeated at least 3 times, as indicated for each experiment in the figure legends.

Supplementary figures

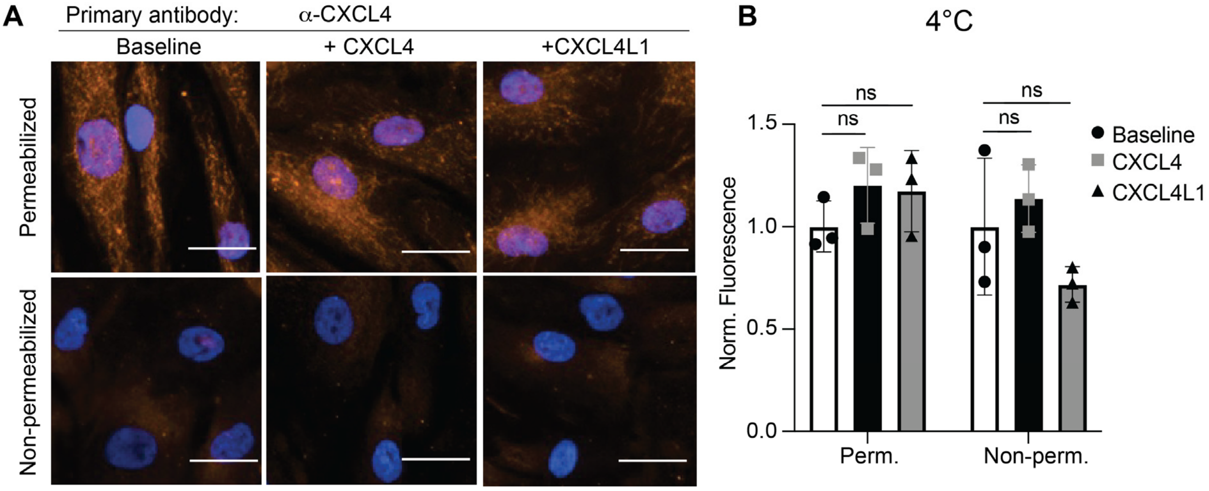

Figure S1: Uptake of CXCL4 into VSMCs.

CXCL4, CXCL4L1 (at 0.5µg/mL) or buffer (baseline) was added to VSMCs for 1 hour at 4°C, washed with heparin and stained for CXCL4 without or with prior permeabilization. **(A)** Representative micrographs showing CXCL4 staining (orange) and nuclei (blue). Scale bar: 50 µm. **(B)** Quantification of fluorescence expressed as normalized fluorescence relative to baseline, measure using Cytation™. ***$P<0.001$, n=3, two-way ANOVA with Dunnett's post-test.

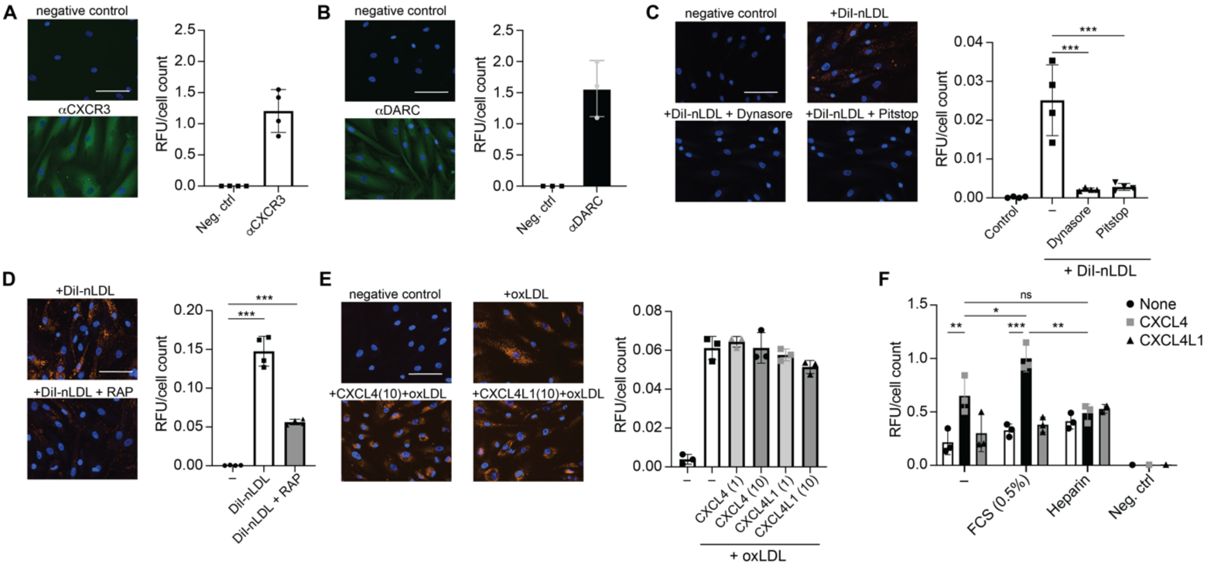

Figure S2: Chemokine receptor staining and molecular determinants of LDL and CXCL4 uptake.

Cells were stained with indicated antibodies or labeled proteins and representative images **(A-E)** are shown (scale bar: 100µm). Fluorescence was quantified and expressed as relative fluorescent units (RFU) divided by cell count **(A-F)**.

Cells were stained with antibodies against CXCR3 **(A)** or DARC **(B)**. DiI-labeled LDL was co-incubated with Dynasore (80 µM) or PitStop2 (15 µM) **(C)** or RAP (200 nM) **(D)**. **(E)** Cells were incubated with (DiI)-labeled oxLDL (5 µg/ml) with or without CXCL4 or CXCL4L1 at 1 and 10 µg/mL. **(F)** Prior to the addition of CXCL4, CXCL4L1 (at 0.5µg/mL) or buffer, VSMCs were cultured in the presence of heparin (200 U/ml) or in DMEM/0.5% FCS for 5 days. After incubation with chemokines for 1 hour at 37°C, cells were washed with heparin and stained for CXCL4 with permeabilization. *P<0.05, **P<0.01, ***P<0.001, n=3-4 independent experiments, one or two-way ANOVA with Dunnett's or Tukey's post-test.

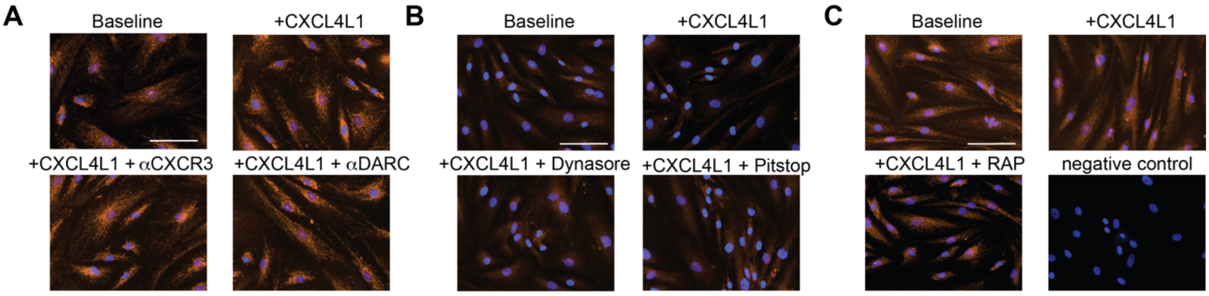

Figure S3. Molecular determinants of CXCL4L uptake. CXCL4L1 (at 0.5 µg/mL) or buffer was added to VSMCs in the presence of indicated compounds for 1 hour at 37°C, washed with heparin and stained for CXCL4 with prior permeabilization. (A) Representative images of CXCL4L1, co-incubated with antibodies against CXCR3 or DARC and resulting intracellular CXCL4(L1) was visualized using Cytation™. (B) Representative images of CXCL4L1, co-incubated with Dynasore (80 µM) or PitStop2 (15 µM) and resulting intracellular CXCL4(L1) was visualized using Cytation™. (C) Representative images of CXCL4L1, co-incubated with RAP (200 nM) and resulting intracellular CXCL4(L1) was visualized using Cytation™.

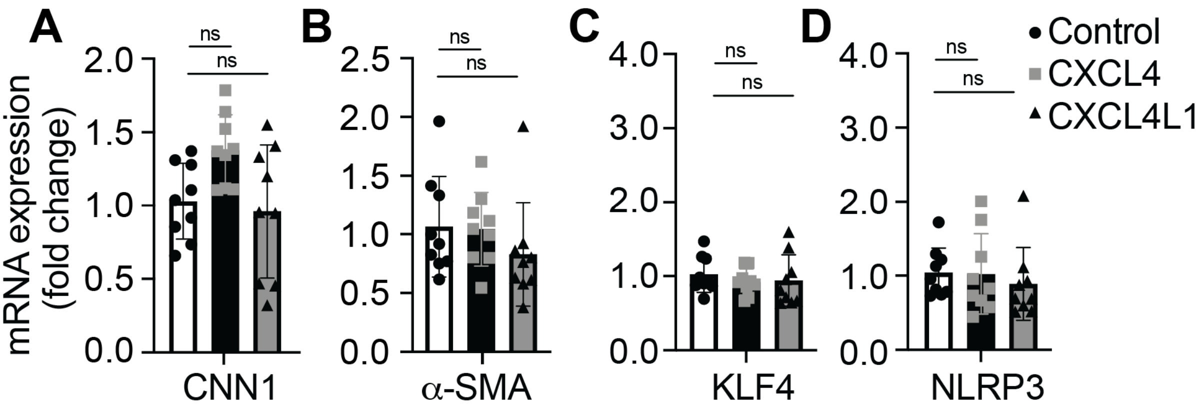

Figure S4: Expression of mRNA by quantitative real time PCR.
Primary VSMCs were cultured at 120.000 cells/well in DMEM with 20% FBS prior to treatment without or with CXCL4 or CXCL4L1 at (1 µg/ml) in DMEM with 2.5% FCS for 72 hours. Relative gene expression was expressed as fold change of **(A)** calponin (CNN1), **(B)** α-smooth muscle actin (α-SMA), **(C)** KLF4 and **(D)** NLRP3. Non-significant, n=7-9 independent experiments, one-way ANOVA with Dunnett's post-test.

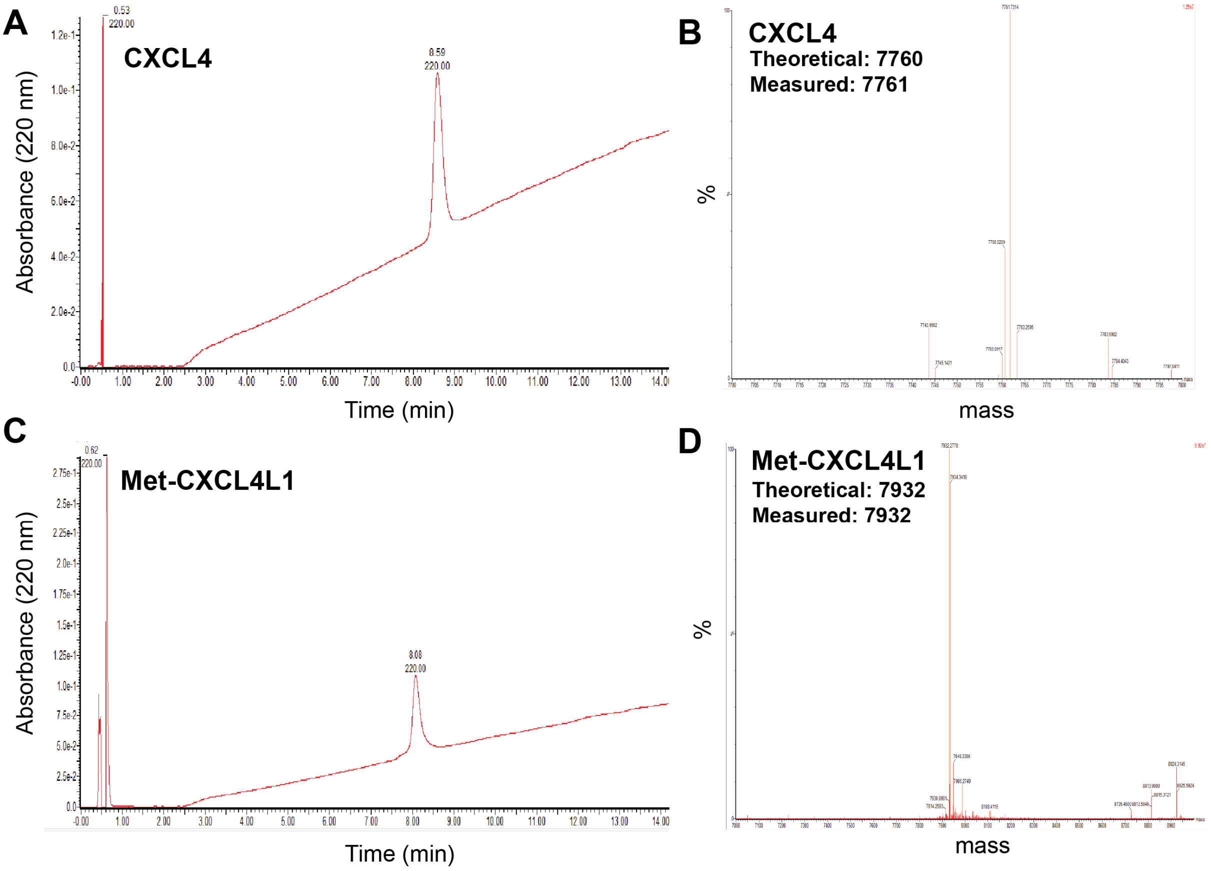

Figure S5: Liquid chromatography–mass spectrometry (LC-MS) analysis of CXCL4 and CXCL4L1

The final pooled CXCL4 and CXCL4L1 fractions from reverse-phase HPLC were analyzed for purity by UPLC-MS. Chromatograms from a C18 column monitored at 220 nm of CXCL4 (A) and CXCL4L1 (B) and deconvoluted mass spectra from CXCL4 (C) and CXCL4L1 (D) with indicated (monoisotopic) theoretical and measured masses. Note: recombinant CXCL4L1 retains its N-terminal methionine, which does not affect its function.

References

1. Struyf, S.; Burdick, M.D.; Proost, P.; Van Damme, J.; Strieter, R.M. Platelets release CXCL4L1, a nonallelic variant of the chemokine platelet factor-4/CXCL4 and potent inhibitor of angiogenesis. *Circ Res* **2004**, *95*, 855-857, doi:10.1161/01.RES.0000146674.38319.07.
2. Sarabi, A.; Kramp, B.K.; Drechsler, M.; Hackeng, T.M.; Soehnlein, O.; Weber, C.; Koenen, R.R.; Von Hundelshausen, P. CXCL4L1 inhibits angiogenesis and induces undirected endothelial cell migration without affecting endothelial cell proliferation and monocyte recruitment. *J Thromb Haemost* **2011**, *9*, 209-219, doi:10.1111/j.1538-7836.2010.04119.x.
3. Kuo, J.H.; Chen, Y.P.; Liu, J.S.; Dubrac, A.; Quemener, C.; Prats, H.; Bikfalvi, A.; Wu, W.G.; Sue, S.C. Alternative C-terminal helix orientation alters chemokine function: structure of the anti-angiogenic chemokine, CXCL4L1. *J Biol Chem* **2013**, *288*, 13522-13533, doi:10.1074/jbc.M113.455329.
4. Agten, S.M.; Suylen, D.; Ippel, H.; Kokozidou, M.; Tans, G.; van de Vijver, P.; Koenen, R.R.; Hackeng, T.M. Chemoselective oxime reactions in proteins and peptides by using an optimized oxime strategy: the demise of levulinic acid. *Chembiochem* **2013**, *14*, 2431-2434, doi:10.1002/cbic.201300598.
5. Groeneweg, M.; Vergouwe, M.N.; Scheffer, P.G.; Vermue, H.P.; Sollewijn Gelpke, M.D.; Sijbers, A.M.; Leitinger, N.; Hofker, M.H.; de Winther, M.P. Modification of LDL with oxidized 1-palmitoyl-2-arachidonoyl-sn-glycero-3-phosphorylcholine (oxPAPC) results in a novel form of minimally modified LDL that modulates gene expression in macrophages. *Biochim Biophys Acta* **2008**, *1781*, 336-343, doi:10.1016/j.bbalip.2008.04.016.
6. Reynolds, J.L.; Joannides, A.J.; Skepper, J.N.; Mcnair, R.; Schurgers, L.J.; Proudfoot, D.; Jahnen-Dechent, W.; Weissberg, P.L.; Shanahan, C.M. Human vascular smooth muscle cells undergo vesicle-mediated calcification in response to changes in extracellular calcium and phosphate concentrations: A potential mechanism for accelerated vascular calcification in ESRD. *Journal of the American Society of Nephrology* **2004**, *15*, 2857-2867, doi:10.1097/01.ASN.0000141960.01035.28.
7. Furmanik, M.; Chatrou, M.; van Gorp, R.; Akbulut, A.; Willems, B.; Schmidt, H.; van Eys, G.; Bochaton-Piallat, M.L.; Proudfoot, D.; Biessen, E.; et al. Reactive Oxygen-Forming Nox5 Links Vascular Smooth Muscle Cell Phenotypic Switching and Extracellular Vesicle-Mediated Vascular Calcification. *Circ Res* **2020**, *127*, 911-927, doi:10.1161/CIRCRESAHA.119.316159.
